# Distinguishing low frequency oscillations within the 1/*f *spectral behaviour of electromagnetic brain signals

**DOI:** 10.1186/1744-9081-3-62

**Published:** 2007-12-10

**Authors:** Charmaine Demanuele, Christopher J James, Edmund JS Sonuga-Barke

**Affiliations:** 1 Signal Processing and Control Group, Institute of Sound and Vibration Research, University of Southampton, Southampton, UK; 2 Developmental and Brain Behaviour Unit, School of Psychology, University of Southampton, Southampton, UK

## Abstract

**Background:**

It has been acknowledged that the frequency spectrum of measured electromagnetic (EM) brain signals shows a decrease in power with increasing frequency. This spectral behaviour may lead to difficulty in distinguishing event-related peaks from ongoing brain activity in the electro- and magnetoencephalographic (EEG and MEG) signal spectra. This can become an issue especially in the analysis of low frequency oscillations (LFOs) – below 0.5 Hz – which are currently being observed in signal recordings linked with specific pathologies such as epileptic seizures or attention deficit hyperactivity disorder (ADHD), in sleep studies, etc.

**Methods:**

In this work we propose a simple method that can be used to compensate for this 1/*f *trend hence achieving spectral normalisation. This method involves filtering the raw measured EM signal through a differentiator prior to further data analysis.

**Results:**

Applying the proposed method to various exemplary datasets including very low frequency EEG recordings, epileptic seizure recordings, MEG data and Evoked Response data showed that this compensating procedure provides a flat spectral base onto which event related peaks can be clearly observed.

**Conclusion:**

Findings suggest that the proposed filter is a useful tool for the analysis of physiological data especially in revealing very low frequency peaks which may otherwise be obscured by the 1/*f *spectral activity inherent in EEG/MEG recordings.

## Background

### The significance of slow waves in literature

Whereas conventional, clinical, EEG recordings consider brain activity within a frequency range of 0.5–50 Hz, several studies have shown that physiological and pathological EEG activity ranges from 0 Hz to several hundreds of Hz. Hence, the emergence of the recent term, full band EEG, which considers both infraslow and ultrafast EEG activity [1]. Ultrafast activity is likely to be apparent in the electrophysiology of neurocognition and motor initiation [2]. Infraslow (ISO) EEG oscillations have been recorded in the EEG of preterm neonates (0.01 to 0.1 Hz) and during epileptic seizure activity [1].

Several studies have concentrated on these ISOs. Some recent work demonstrates large amplitude oscillations in the human cortex ranging from 0.02 to 0.2 Hz during sleep [3,4]. Here, each slow wave is accompanied also by an increase in EEG synchronisation between different scalp areas. These ISO fluctuations in EEG synchrony are characterised by the stable dynamical structure of sleep [5]. The work by P. Achermann et al. in [6] illustrates the presence of distinct slow-wave components below 1 Hz in human sleep and the work by I.G. Campbell et al. in [7] suggests that the physiological and molecular mechanisms of these very low frequency EEG activity differ from those of higher frequency bands such as the delta band (1–4 Hz). Supra-second oscillations have been recorded also in the thalamus and basal ganglia of rats, in the cerebral cortex of cats [8], and in the monkey visual cortex [9].

Moreover, the study for the classification of neuronal oscillations in the mammalian cortex in various frequency bands suggests that there are spontaneous coherent low frequency neuronal oscillations within a neuro-anatomically robust default network of brain activity [8]. This has been reinforced by further work by Buzsaki et al. in [9] and M. Steriade et al. in [4] where studies have been carried out attempting to link neuronal activity to behaviour.

All this suggests that the basic rhythms of the EEG (*δ*, *α*, *β*, *γ*) represent only a part of the measured brain activity. Very low frequency oscillations are a salient feature of the EEG and can give access to new insight into brain function.

### Issues in recording and analysing very low frequency EEG activity

Infraslow signal recording requires genuine DC-coupled amplifiers with high input impedance, high DC stability and a wide dynamic range. Sufficiently stable electrodes and adequate gels should be used to provide a faithful EEG recording at such low frequencies [1]. DC drift which is superimposed on any meaningful event-related slow activity can be an issue unless amplifiers are reset every three minutes [10] to ensure that the signal is kept in the optimal range of the amplifier throughout the recording.

Analysing low frequency oscillations may be made more complex by the fact that EEG activity belongs to a broad class of physical signals which arise from a so-called 1/*f *process. Such signals have a power law relationship of the form:

Sx(f)=constant|f|γ
 MathType@MTEF@5@5@+=feaafiart1ev1aaatCvAUfKttLearuWrP9MDH5MBPbIqV92AaeXatLxBI9gBaebbnrfifHhDYfgasaacPC6xNi=xI8qiVKYPFjYdHaVhbbf9v8qqaqFr0xc9vqFj0dXdbba91qpepeI8k8fiI+fsY=rqGqVepae9pg0db9vqaiVgFr0xfr=xfr=xc9adbaqaaeGacaGaaiaabeqaaeqabiWaaaGcbaGaem4uam1aaSbaaSqaaiabdIha4bqabaGccqGGOaakcqWGMbGzcqGGPaqkcqGH9aqpjuaGdaWcaaqaaiabdogaJjabd+gaVjabd6gaUjabdohaZHqaciab=rha0jab=fgaHjab=5gaUjabdsha0bqaamaaemaabaGaemOzaygacaGLhWUaayjcSdWaaWbaaeqabaacciGae43SdCgaaaaaaaa@450B@

where *S*_*x*_(*f*) is the power spectral density, *f *is the frequency and *γ *is some spectral parameter which is usually close to 1 but can lie in the range 0 <*γ *< 2 [11], and could be greater than 2 in the presence of noise sources.

This 1/*f*^*γ *^spectral behaviour was first reported in 1925 in an electric current passing through a vacuum tube [12]. It appears also in economic and communication systems, in electronic transistors and diodes, in the annual amount of rainfall and in the rate of traffic flow [11]. Biological data such as the potential measured across nerves and physiological systems such as the cardiovascular and respiratory mammalian systems also exhibit this kind of behaviour [13].

**Figure 1:**
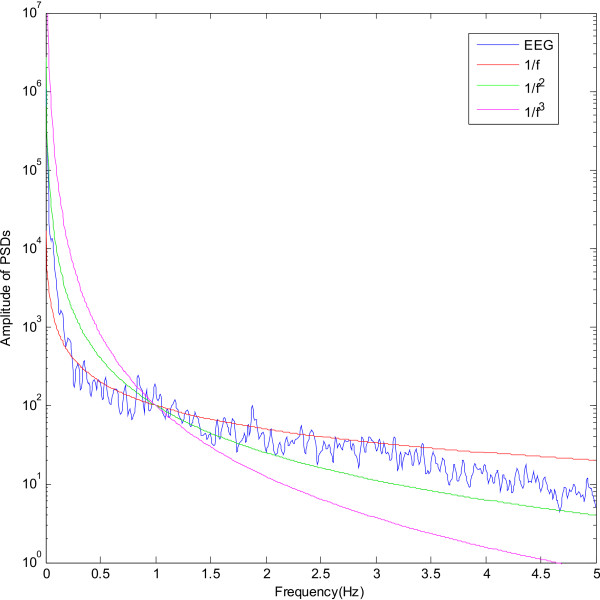
The power spectral density of a typical EEG channel with superimposed 1/*f*^*γ *^curves.

The work in [14] by Kobayashi et. al in 1982 demonstrates that the human heartbeat period fluctuation has this kind of power spectral density for frequencies below 2 × 10^-2 ^Hz but the reason for this behaviour is not known. This 1/*f *fluctuation has also been observed in the body sway motion and in eyeball motion [14]. Over the years, numerous studies have acknowledged that this 1/*f*^*γ *^trend is intrinsic in the neuronal system. The power-law scaling in the brain shows a decrease in log power with increasing frequency, following a 1/*f*^*γ *^curve (Figure 1). This has been observed in the temporal and spatial power spectral densities (PSDs) of EEG recorded both intracranially and on the scalp [15-17].

### The Origin of the 1/*f*^*γ *^spectral behaviour

The inverse relation of the power density of the EEG with frequency in the mammalian cortex is a result of the physical structure of neuronal networks and the limited speed of neuronal communication arising from axon conduction and synaptic delays [9]. A large cluster of neurons, each generating a unit activity, forms a functional network which is held together by the neurons' synchronisation that ensures activity control [18]. Such synchronised behaviour seems to attract further neurons and causes the oscillation amplitude to increase. Moreover, the period of oscillation is determined by the size of this neuronal cluster that constitutes a given cycle [9]. Thus, large neuronal areas are associated with slow, high amplitude oscillations whereas a small, localised concentration of neurons gives rise to higher frequency, low amplitude signals [8]. This explains why most of the power of the EEG signals is concentrated in the low frequency spectrum.

MEG recorded data, which is sometimes preferred over EEG recordings due to its high spatial resolution and the extremely high temporal resolution, also exhibits this 1/*f*^*γ *^behaviour inherent in its power spectrum. This is to be expected since these two systems share the same underlying model – MEG measures the minute magnetic field generated by the electrical activity of neurons. This electrical activity corresponds to that detected by EEG electrodes.

In order to be able to analyse any spectral activity superimposed on this 1/*f *trend the EEG/MEG power spectrum can be normalised by removing this trend [9]. Makinen et al. in [19] employ a technique called Partition-Referenced Moment and use it with wavelet transforms to obtain a level base spectrum. This is used for examining ongoing oscillations and auditory event-related brain processes recorded by MEG. Apart from being an involved approach, the proposed method is based in the frequency domain thus destroying the phase information of the raw signal.

In this paper we describe two methods that can be used to achieve spectral normalisation, i.e. removal of the intrinsic 1/*f*^*γ *^to provide a flat spectral base onto which event-related brain activity is superimposed. The first method is based in the frequency domain – its main aim being to investigate the spectral characteristics of electrophysiological signals and to provide us with a basis for normalisation. The second method is a time domain approach where spectral normalisation is achieved by filtering the raw signals prior to further data analysis. This is a simple and effective method which conserves the phase information of the input signal. For these reasons, we use it on real electrophysiological data, namely epileptic seizures, very low frequency EEG recordings, MEG recordings and Evoked Responses to illustrate its function.

## Methods for spectral normalisation

### A. Normalisation in the frequency domain

Normalisation of the spectrum can be achieved in the frequency domain by dividing any EEG spectrum by an established background 1/*f*^*γ *^spectrum. This concept was tested on the multi-channel EEG recordings of two participants. 32 EEG channels were used and these included ear references at Tp9 and Tp10, two electrooculogram (EOG) channels that monitored the linked vertical and horizontal eye movement, and one electrocardiogram (ECG) channel. Electrode placement was in accordance to the 10–20 system and an electrode cap was used. Impedance levels were set at less than 5 kΩ.

No filters were switched on during the recordings such that DC activity could be captured and DC-stable sintered electrodes were used. The data was sampled at 250 Hz and was digitally stored in a 12 bit ADC. Before analysis the data was decimated by a factor of 25 – this was necessary to ensure an adequate number of samples for the analysis of very low frequencies. Detrending was then carried out to remove the mean shift (over 5 or 10 minutes) in each recording.

In these recordings, every participant followed a ten minutes driving task – where the participant was meant to trail a plain winding track on screen by pressing the arrow buttons on the keyboard. This was followed by five minutes, eyes-closed resting condition during which the participant was seated on a reclining chair. A ten minute arrows task followed, during which the participant was asked to press a button whenever the arrow appearing on screen pointed left or right (according to the instruction given). The participant's recording was concluded by a five minutes eyes-open resting period, again seated on a reclining chair. For every participant these segments of data were analysed separately. The expectation is that under this type of mental load which requires the participants' attention there is predominant low frequency activity around 0.1 Hz. More information behind this can be found in [20].

**Figure 2:**
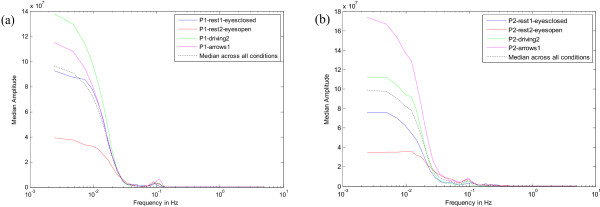
**Median PSD curves for participants 1-(a) and 2-(b)**. Median PSDs showing the 1/*f*^*γ *^relation of the EEG in all conditions. For each participant, the median across the 4 conditions forms the normalisation curve(-).

For a particular participant, the spectrogram of one EEG channel was calculated. The median across all time windows was then found for every frequency point. Thus, a graph of the median PSD value for every frequency was obtained. The same procedure was repeated for all the EEG channels. Then, the overall median of the median PSD curves of all the channels was calculated. The same was done for each of the 'task' and 'rest' condition. The average was then calculated across all conditions and this was used as our normalisation curve (black dashed curves in Figure 2). The reason for considering all channels and all conditions to obtain this curve was to be able to establish a general base picture of the underlying brain activity that gives rise to this 1/*f*^*γ *^distribution. The spectrogram of the EEG data to be analysed, (e.g., Participant 1, driving task), was then calculated and each time window of the spectrogram was divided by this normalisation curve, (in the frequency domain).

**Figure 3:**
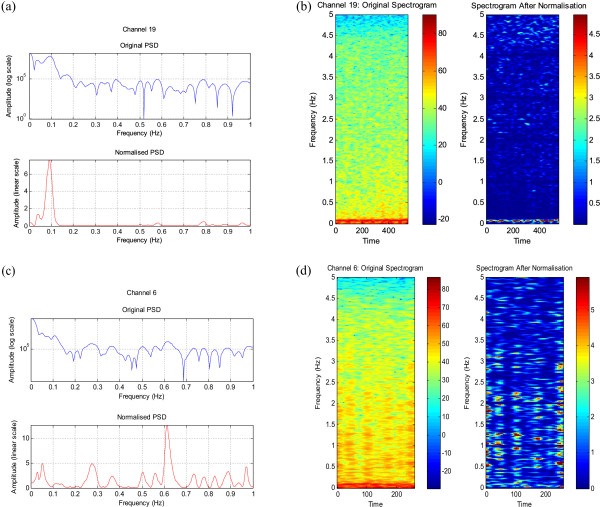
**Normalisation in the frequency domain**. (a) Patient 2 Driving task, (b) Corresponding spectrograms, (c) Patient 1 Eyes-open condition, (d) Corresponding spectrograms.

From the plots in Figure 2 the 1/*f*^*γ *^nature of the EEG can be observed in all median curves. This gives us confidence in the current method of estimation of the normalisation curve. The plots in Figure 3 show the normalised spectrum – the the 1/*f*^*γ *^trend is removed and event-related peaks can be easily distinguished from ongoing brain activity. A peak at 0.1 Hz can be clearly seen in Figure 3(a) and this is manifested in the normalised spectrogram of Figure 3(b). In Figure 3(c) no appreciable low frequency peaks can be seen and Figure 3(d) shows the normalised spectrogram with no prominent low frequency activity.

### B. Time domain spectral normalisation approach

This type of normalisation can be achieved by passing the EEG input signal through a filter that cancels the 1/*f*^*γ *^spectral behaviour prior to any signal analysis. This inverse filter can be established by modelling the normalisation curve shown in Figure 2 by an Autoregressive (AR) or a Moving Average (MA) model and then swapping the coefficients to obtain its inverse. Hence,

*A*/*f*^*γ *^× *Bf*^*γ *^≈ *AB*

where *A*/*f*^*γ *^is the EEG spectrum with the intrinsic 1/*f*^*γ *^characteristics, *Bf*^*γ *^is the inverse filter contribution and AB is the result of their interaction, implying that the output is a normalised spectrum. The 1/*f*^*γ *^curve can be modelled as a finite impulse response (FIR) model such that its inverse will be an infinite impulse response (IIR) model. However the problem is the lack of control on the FIR coefficients since these are already predetermined by the shape of the normalisation curve. Thus if the resultant FIR model is not minimum phase the IIR model stability becomes a major issue. Moreover we require the filter to be a linear phase filter to avoid phase distortion of the input EEG signal – and an IIR filter will not meet this requirement.

**Figure 4:**
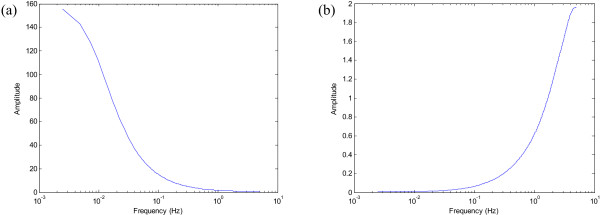
**Modelling the inverse filter**. (a) An estimate of the normalisation curve showing a 1/*f*^*γ *^frequency response obtained by a 6th order AR model, (b) The inverse filter frequency response obtained from the corresponding 6th order MA model.

Another possible approach is that of modelling the normalisation curve as an AR model such that its inverse is an MA model and stability is guaranteed. The time domain representation of the normalisation curve obtained in the frequency domain is found by computing its Inverse Fourier Transform. The AR coefficients of an IIR filter are then obtained using the Yule-Walker equations on the absolute value of this time domain signal. The AR frequency response provides an estimate of the normalisation curve as shown in Figure 4(a). Swapping the coefficients of this model provides us with an MA (FIR) filter which has a frequency response that is the inverse of the estimate of the normalisation curve, (Figure 4(b)).

**Figure 5:**
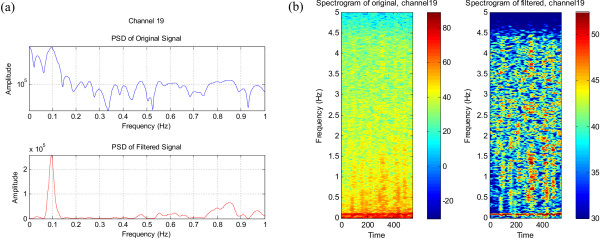
**Time domain spectral normalisation by ARMA modelling**. (a) Spectral normalisation achieved by the input FIR filter, (b) The corresponding spectrograms of the original EEG signal (amplitude in log scale) and of the filtered signal (amplitude in linear scale) showing a dominant peak at 0.1 Hz.

Although this approach gives the expected results (as shown in Figure 5), it is an involved method since it uses the normalisation curve in the frequency domain in order to derive the appropriate inverse filter.

#### Approximating the inverse filter by a differentiator

Figure 1 shows 1/*f*^*γ *^curves superimposed on the power spectral density of a typical EEG channel, with *γ *varying from 1 to 3. It is clear that the 1/*f *curve follows closely the EEG spectral trend across the entire frequency band. This was verified for a number of participants under different task and rest conditions. Therefore the normalisation curve can be approximated to be a 1/*f *curve, i.e. setting *γ *= 1, and the inverse filter can be obtained by applying a differentiator. This could be done using the **cfirpm **function in MATLAB, which provides a set of filter coefficients that simulate a linear phase differentiator.

Alternatively, the differentiator can also be modelled as a 2nd order MA filter. A differentiation function is given by:

y(t)=x(t)−x(t−1)Δt
 MathType@MTEF@5@5@+=feaafiart1ev1aaatCvAUfKttLearuWrP9MDH5MBPbIqV92AaeXatLxBI9gBaebbnrfifHhDYfgasaacPC6xNi=xI8qiVKYPFjYdHaVhbbf9v8qqaqFr0xc9vqFj0dXdbba91qpepeI8k8fiI+fsY=rqGqVepae9pg0db9vqaiVgFr0xfr=xfr=xc9adbaqaaeGacaGaaiaabeqaaeqabiWaaaGcbaGaemyEaKNaeiikaGIaemiDaqNaeiykaKIaeyypa0tcfa4aaSaaaeaacqWG4baEcqGGOaakcqWG0baDcqGGPaqkcqGHsislcqWG4baEcqGGOaakcqWG0baDcqGHsislcqaIXaqmcqGGPaqkaeaacqqHuoarcqWG0baDaaaaaa@413C@

where x(t) is the input signal, y(t) is the filtered output and Δ*t *= *T*_*s *_= 1/*f*_*s*_, *f*_*s *_being the sampling frequency. Thus the MA filter coefficients can be set as 1/Ts and -1/Ts.

#### Differentiator characteristics

**Figure 6:**
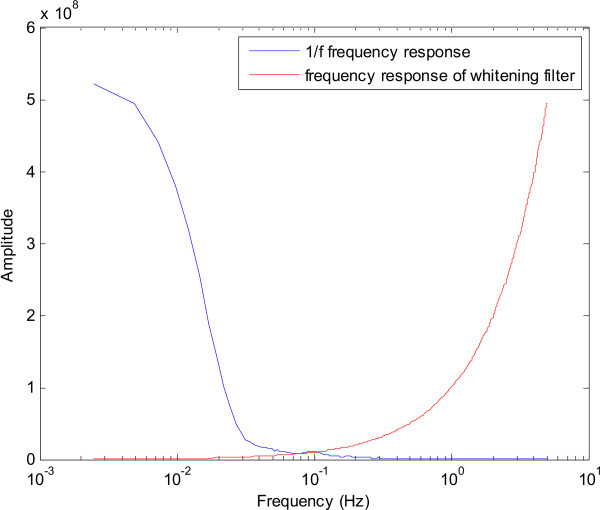
The function of the differentiator in spectral normalisation.

**Figure 7:**
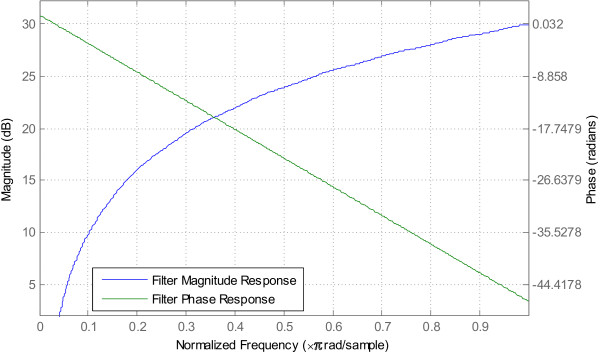
Magnitude and phase response of the differentiator.

The differentiator, with its *f *frequency response, cancels out the 1/*f *trend of the EEG power spectrum as described in equation (2). This is illustrated in Figure 6. Figure 7 shows the magnitude and phase response of the differentiator. The filter exhibits a linear phase response and a constant group delay. The linear phase response makes it easier to compensate for the phase delay at one particular time instant by sample-shifting the pre-recorded data accrodingly.

**Figure 8:**
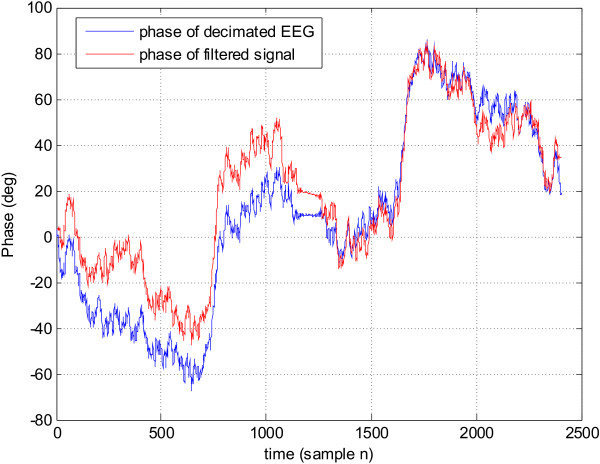
The phase effect of the differentiator on the input EEG signal.

The effect of the filter on the phase of the input EEG signal can be seen in Figure 8. Although the phase difference between the input and the output of the filter is not constant across time due to variation in the signal freqeuncy content, the general shape of the phase is preserved. Moreover since the phase response of the filter is known and fixed, any input signal will experience the same phase delay at one particular frequency. Consequently if one is interested in establishing the phase synchronisation between two channels (which is computed at one specific narrow frequency band), the phase relationship of the two signals will not be distorted by the filter since both signals will be delayed by the same amount at that frequency.

This analysis shows that performing normalisation in the time domain by using a differentiator is a very viable approach. This is because, although the frequency domain method produces a normalised spectrum without assuming that the underlying bias is a strict 1/*f *curve, (since the normalisation curve is modelled directly from the dataset), the phase information of the EEG signal is lost. Consequently, it is not possible to reconstruct the time-domain series from the normalised spectrum. Moroever the resultant time series cannot be used to extract its phase interactions with other normalised data. For these reasons, the differentiator is used here to filter different datasets in order to demonstrate its function as a tool for spectral normalisation. The results are illustrated in the following section.

## Results

### Applying the differentiator to various datasets

#### Synthetic data

The differentiator was applied to two sinusoidal signals of frequencies 0.1 Hz and 0.5 Hz respectively, superimposed on normal background EEG. The signal to noise ratio (SNR) of the higher frequency signal (SNR2) was kept fixed at 15 dB whereas that of the lower frequency signal (SNR1) was varied from 0 to 47 dB. Each SNR was measured by calculating the ratio of power of the sine wave to that of the background EEG.

**Figure 9:**
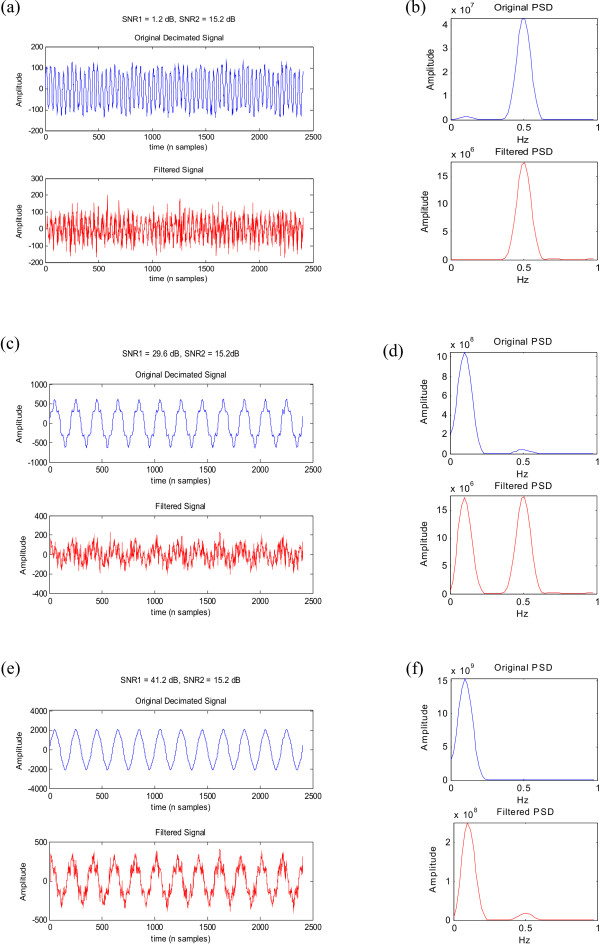
**Time domain signal consisting of 2 sine waves at 0.1 Hz and 0.5 Hz of varying SNR (SNR1 and SNR2 respectively) filtered through the differentiator and their corresponding spectra before and after filtering**. (a) SNR1 is less than SNR2, (c) SNR1 is approximately twice SNR2, (e) SNR1 is much higher than SNR2; (b), (d) and (f) show the corresponding spectra.

When SNR1 is less than SNR2 (Figures 9(a) and 9(b)) the filter attenuates the low frequency component significantly by removing the 1/*f *trend. In the second case (Figures 9(c) and 9(d)) the magnitude of the 0.1 Hz filtered component becomes equal to that of the 0.5 Hz component since SNR1 is high enough to compensate for the 1/*f *intrinsic spectral behaviour. This means that the SNR of the lower frequency component needs to be twice that of the higher frequency component to place it above the 1/*f *curve and let it be apparent at the output. Any SNR1 exceeding this threshold results in the low frequency component being higher than the high frequency component after filtering (Figures 9(e) and 9(f)).

**Figure 10:**
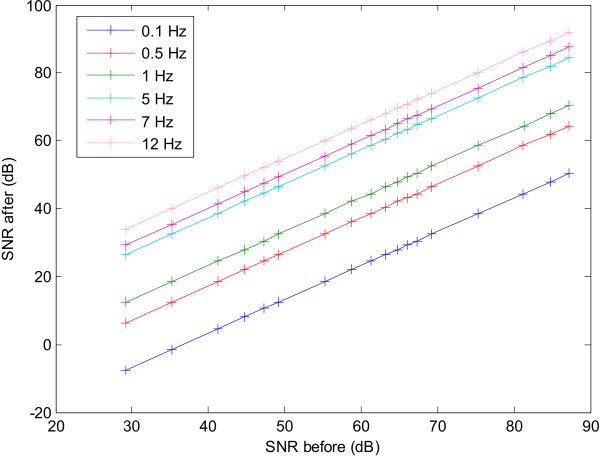
SNR before and after filtering for various input frequencies of a sine wave used as an input signal to the differentiator.

**Figure 11:**
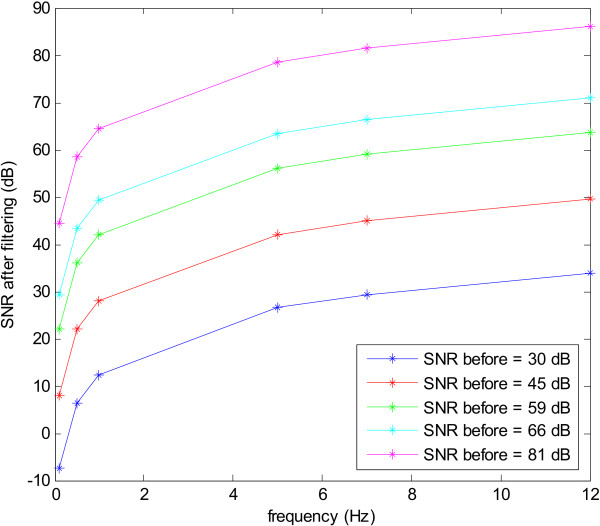
Variation across frequencies of the output SNR for fixed SNRs of the input signal.

Figure 10 shows the SNR for an input sine wave as its frequency is varied from 0.1 Hz to 12 Hz. It is clear that for every input frequency the SNR before filtering varies linearly with that after filtering. Moreover lower frequencies have a lower SNR after filtering due to the 1/*f *base spectrum. This is shown in Figure 11, where for a particular SNR before filtering, the SNR after filtering increases as the frequency of the input signal becomes higher. The curves in this figure can be approximated by an inverse 1/*f*, hence implying that the differentiator is attenuating lower frequencies more than higher frequencies hence compensating for the 1/*f *bias.

#### Epileptic seizure data

Focal epileptic seizure data recorded using twenty-five electrodes placed on the scalp according to the International 10–20 electrode placement system with reference at FCz, was used as input to the differentiator. The three minute long data recording was sampled at 200 Hz and digitally stored at 12 bit resolution. The recording included pre-ictal, ictal and post-ictal activity. The seizure was focused at the left-temporal lobe (around T3).

When considering seizure data the relationship between SNR before and after filtering shows the same linear trend like that obtained for synthetic data. Here, the SNR was loosely computed by finding the ratio of power of the three minute EEG data incorporating seizure activity to the power of ongoing background EEG activity of the same duration recorded for the same subject. This procedure was carried out on the data before and after passing it through the differentiator.

**Figure 12:**
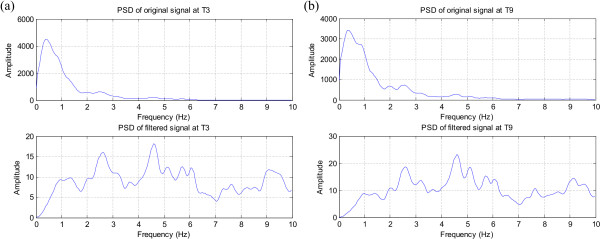
**The effect of the filter on the spectrum of the signals recorded around the seizure focus**. (a) T3 signal spectrum, (b) T9 signal spectrum; Note the removal of the 1/f trend and the clear peak around 4.5 Hz indicating the rhythmic seizure activity.

**Figure 13:**
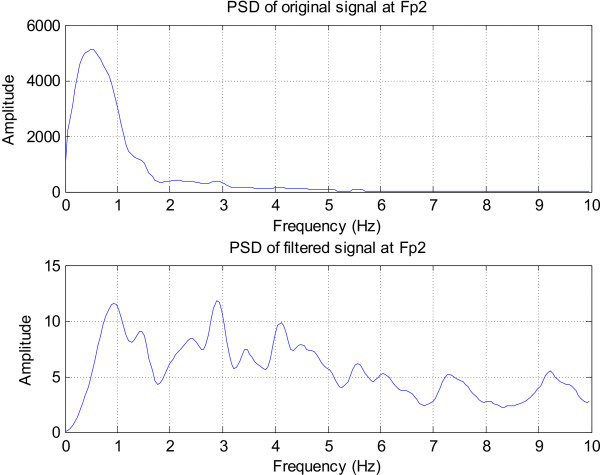
**The spectra of Fp2 (channel with eye movement activity)**. Eye movement activity has lower frequency peaks which are more pronounced in the normalised spectrogram.

Figure 12 and Figure 13 show the effect of filtering on the spectrum of selected EEG channels. After filtering the original spectra in Figure 12(a) and (b) are flattened and the 1/*f *trend is clearly removed. Moreover, the peak around 5 Hz, which is related to the rhythmic seizure activity becomes much more pronounced in the filtered spectra. The spectrum of the frontopolar channel (Fp2) in Figure 13 is also normalised by the differentiator and low frequency peaks due to eye-related activity become clearly visible.

#### EEG data with LFO activity

**Figure 14:**
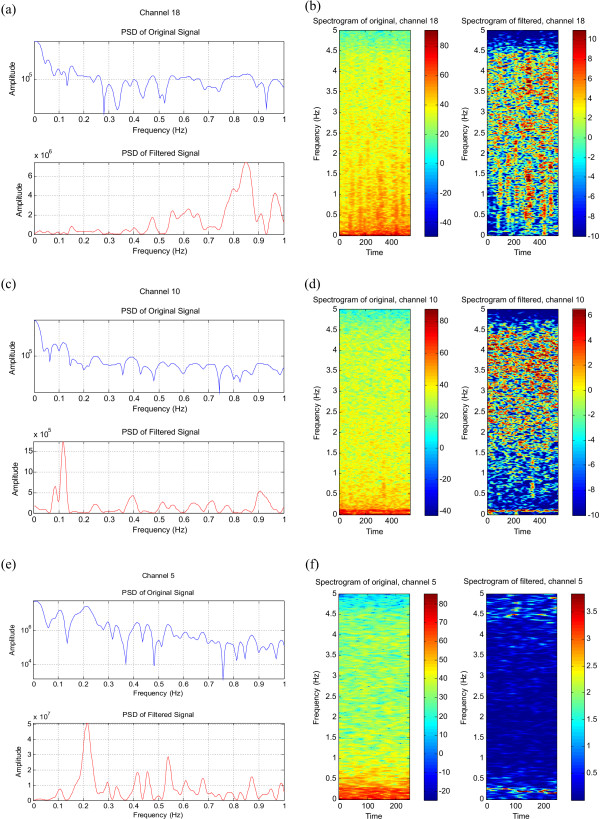
The normalised spectra (a), (c) and (e), and the corresponding spectrograms (b), (d) and (f) of the EEG signals with LFO's.

When the same EEG data used in the 'Normalisation in the Frequency Domain' section of this work was applied to this filter the spectrum was normalised as expected. No significant low frequency activity can be seen in the spectrum of the filtered signal in Figure 14(a), whereas dominant 0.1 Hz activity is visible in the normalised spectrum of Figure 14(c). A prominent LFO at 0.2 Hz, which has been obscured by the ongoing spectral activity prior to filtering, can be clearly seen in Figure 14(e). The corresponding normalised spectrograms are shown in Figures 14(b),(d) and 14(f) respectively.

Thus the resultant spectrogram obtained by normalising in the frequency domain and that obtained by normalising in the time domain are very similar – they both show a peak at 0.1 Hz (or 0.2 Hz) and a flat spectrogram when no extra low frequency activity is expected. One can note similar activity around 0.1 Hz in Figure 3(b) (normalisation in the frequency domain) and Figure 14(d) (normalisation in the time domain) for the same data set.

#### MEG data

Another useful application of the differentiator is in the analysis of MEG data. MEG, the recording of the magnetic activity of the brain is measured in a whole-head system. A CTF Systems 151 channel MEG system was used to record over 20 minutes of ongoing activity in a normal, healthy volunteer. The examples given here demonstrate that passing MEG data through this type of filter achieves normalisation of its spectra.

**Figure 15:**
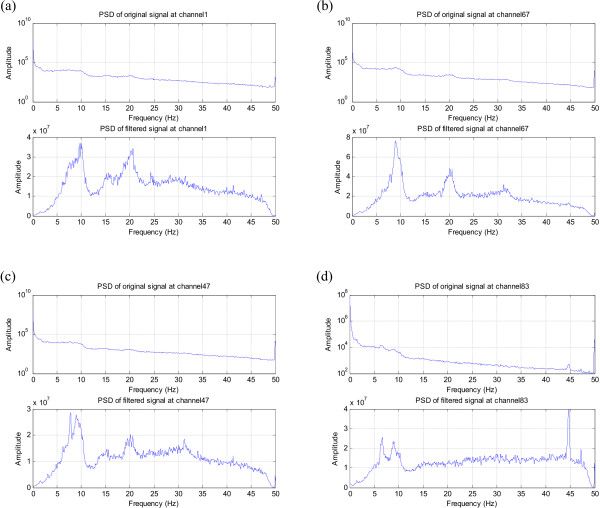
**Normalised spectra of selected MEG channels**. Note the prominent peaks in the PSDs of the MEG-recorded filtered signals, which were previously obscured by the 1/f trend in the PSDs of the raw unfiltered signals.

The data was downsampled to a sampling rate of 100 Hz, and was used as the input of the differentiator. The original and the normalised spectra of some of these channels are shown in Figure 15. The peaks around 10 Hz and 20 Hz in Figures 15(a) and 15(b) are barely visible in the original PSD due to the 1/*f *trend but become very evident after filtering. In Figure 15(c) and 15(d), the distinct low frequency components around 7 Hz and 9 Hz are much more pronounced in the normalised spectrum. Moreover, the filtered spectrum in Figure 15(d) shows that the peak at 44 Hz has a higher amplitude relative to the lower frequency components once the 1/*f *trend is compensated for. This shows the importance of this technique for clearly distinguishing prominent frequency components as well as for comparing signal power in different frequency bands.

#### Evoked response data

Another relevant set of EEG signals are those involving evoked responses. The data set used here contains P300 evoked potentials. One minute's worth of EEG data sampled at 240 Hz was used and the analysis was focused on electrodes C3, Cz and C4 where the P300 response was expected. The participant was presented with a six by six matrix of characters. The task was to focus attention on characters in a word that was prescribed by the investigator (i.e. one character at a time). The data contained 35 epochs of 1.5 second duration each. The P300 stimulus appeared at 0.5 seconds in every epoch so the P300 response was expected to occur at 0.8 seconds within the epoch.

**Figure 16:**
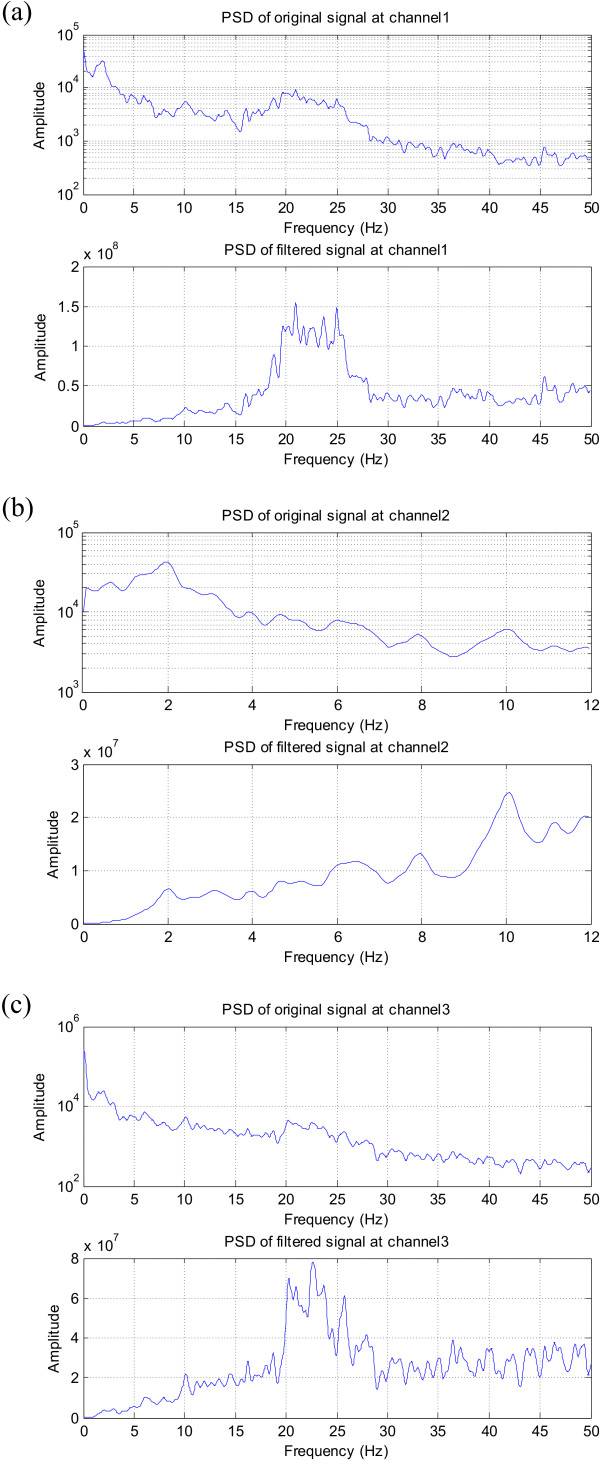
**Evoked responses data normalised to reveal spectral peaks**. (a) PSD of Channel C3, (b) PSD of Channel Cz, (c) PSD of Channel C4. Note the low peak apparent at 10 Hz corresponding to a weak P300 response in (b) and the clear prominent activity in the gamma band around 20–30 Hz in (a) and (c).

On computing the PSD of the three EEG signals, it became evident that these also exhibit 1/*f *spectral behaviour. This can be expected because evoked responses share the same physical model as the EEG in that they both involve the recording of the brain electrical activity – the only difference being that evoked potentials are time-locked to a stimulus and generally have lower amplitudes than EEG. Filtering can thus be used to normalise their spectra for clearer data analysis as shown in Figure 16.

## Discussion

In this paper the importance of spectral normalisation has been emphasised as a way of revealing event-related peaks which may otherwise be obscured by the intrinsic 1/*f *spectral activity in EM brain signal recordings. Because of this 1/*f *trend EM brain signals have spectra with high power at low frequencies. Normalisation renders a flat base spectrum when no extra low frequency activity is present and reveals distinct peaks related to specific cognitive tasks or mental conditions. This is particularly important for the analysis of very low frequency oscillations (0.05 – 0.5 Hz) apparent in EEG and/or MEG signals. Frequency domain normalisation destroys the phase information of the input signal and excludes the possibility of signal reconstruction after spectral whitening. In this work we propose a time domain approach which employs a differentiator to cancel the 1/*f *trend. This is a simple solution which does not require the selection of various parameters. Moreover, the filter leaves the signal phase intact due to its linear phase characteristics. Here we have shown its application in a broad range of physiological signals including epileptic seizure data, EEG data with very low frequency characteristics, MEG data and evoked response recordings. In each case, the spectral normalisation helped to highlight peaks of interest across the spectra.

## Conclusion

Spectral normalisation is achieved in all of the mentioned datasets. This implies that:

• The underlying background electrophysiological activity does indeed follow a 1/*f *trend

• The approximation to model the inverse of the normalisation curve by a differentiator is suitable.

Future work involves the use of this filter for the analysis of data that has very low frequency activity that is of interest. This technique can also be used for comparison of power between the very low frequency and the conventional or higher frequency bands during different cognitive processes. Its linear phase response makes it possible to investigate the phase synchronisation between pairs of filtered data at those frequency bands where prominent peaks appear in the normalised spectra.

## Competing interests

The author(s) declare that they have no competing interests.

## Authors' contributions

All authors have read and approved the final manuscript.
